# The exploration of size and toddler interaction with liquid laundry detergent capsules

**DOI:** 10.1371/journal.pone.0244481

**Published:** 2020-12-30

**Authors:** Annalise Richmond, David C. Schwebel, Casie H. Morgan, Zhiwu W. Liang, Alice Boutoille, Pablo Buso, Ana Mata, Gerard Stijntjes

**Affiliations:** 1 Procter and Gamble, Strombeek Bever, Belgium; 2 Department of Psychology, University of Alabama at Birmingham, Birmingham, Alabama, United States of America; 3 Aiju AIJU, Technological Institute for children’s products & leisure, Alicante, Spain; University of Campinas, BRAZIL

## Abstract

Liquid laundry capsules have been involved in multiple poisoning incidents with young children in the home. There are a range of contributing factors for these incidents, including influences from industry, culture, home environments, and parenting/supervision. There also are influences from children’s behaviour and decisions in reaction to potential hazards. Previous research examined the influence of capsule product appearance and colour on children’s behaviour around hazardous household items, but little research examines the influence of product size. This research explored if differences in the size of liquid laundry capsules result in different levels of toddler interaction. We compared two commercially available capsule designs that are identical in physical appearance but differ in physical size. Our research was conducted using three studies: Study 1, forced-choice test in an out-of-context laboratory setting; Study 2, an ecologically-valid, simulated real-world setting replicating a home laundry cabinet with a container of capsules left open; and Study 3, a second ecologically-valid study replicating a home laundry cabinet, this time with a capsule left outside its container. Capsule interaction was measured by grasping choice among samples of 156 toddlers ages 9–36 months. The same sample was used for Studies 1 and 2, and a second identically sized sample recruited for Study 3. Results from Study 1 indicated toddlers selected the small (49.8% selection) and large (50.2%) capsule with nearly identical frequency. Study 2 largely replicated Study 1: Toddlers selected the small capsule or container of small capsules 26.8% of the time and the large capsule or container of large capsules 22.3% of the time. Study 3 also replicated previous findings: Toddlers selected the smaller capsule 18.0% of the time and the larger 19.2%. We discuss study results, which suggest no appreciable difference in toddler’s grasping choice to smaller versus larger laundry capsules.

## Introduction

Scholarly research highlights safety concerns with liquid laundry capsules, with some reports suggesting the physical appearance of the laundry capsules may contribute to child health risk because toddlers may touch and mouth them, leading to unintentional poisoning incidents [1–5]. The highest risk of accidental exposure occurs among toddlers ages 9–36 months, with about 75% of total reported incidents among children in the US falling within this age range [3–8].

Given these concerns, it is important to understand factors that may contribute to toddler interaction with laundry capsules. From a conceptual and theoretical perspective, one might cite theories explaining that multiple interactive and overlapping factors contribute to individual injury events like poisoning incidents [9–11]. Bronfenbrenner’s ecological model [11], for example, proposes that various aspects of a child’s environment interact with that child to influence decisions and behaviour. There are concomitant and overlapping influences from broader macrosystem influences like the family’s culture (e.g., risk-taking tendencies, how laundry areas are typically arranged in a home) and from industry (e.g., packaging and labelling of products), as well as from more proximal microsystems like the family environment (e.g., parent supervision habits). Consumer misperceptions about the risk of products like laundry detergents also play a role in the microsystem, creating a situation where parents may not fully appreciate the risk of poisoning to their children and therefore implement insufficient safeguards and storage habits [12–16].

There also are risk factors that emerge at the individual level in Bronfenbrenner’s ecological model; these are factors that emerge from the children’s own decisions and behaviours. Research in related domains suggests children may interact with different household products at different rates based on products’ appearance–including their shape, colour and transparency; these factors could therefore influence children’s risk of unintentional poisoning [17–22]. Research suggests the shape and colour of the product may be especially salient in young children’s categorization of edible and non-edible products [17,21,22].

There is limited research focused specifically on how children may interact with laundry capsules. Most published research focuses on macrosystem-level influences by examining laundry capsule poisoning outcomes, exposure routes, and management of laundry capsule exposure [23,24] rather than individual-level factors related to toddler’s interaction with the capsules. One recent study found mixed results in toddlers’ interaction with different multi-coloured and mono-coloured laundry capsules [25]. In a forced-choice laboratory test, toddlers ages 12–36 months showed some preference toward multi-coloured rather than mono-coloured capsules. In a more ecologically valid real-world replication of a home environment, however, no significant differences in toddler’s interactions emerged [25]. In support of these findings, a review of child exposures reported to US Poison Control Centres found no significant differences between children’s interaction with clear versus coloured laundry capsules [26].

The present paper focuses not on the influence of laundry capsule colour on children’s interactions with them, but on the impact of a different physical characteristic, capsule size. Over the past two decades, household washing machines have become larger, the result of two concurrent patterns. First, many consumers prefer to wash clothes less frequently but with larger loads, seeking efficiency and ease of completing household laundry tasks amidst their busy schedules [27]. Second, regulations in the United States have incentivized movement toward higher-capacity, high-efficiency washing machines on the market, explaining: “Products with a larger capacity are inherently able to achieve higher efficiency levels” [28]. These patterns have resulted in washing machine capacity growing over 30% between 1999 and 2015, with 52% of laundry loads completed in the US classified as “large” load machines (roughly 12–13 lbs. [5.44–5.90 kg]) [28,29]. To properly clean their larger loads of laundry, consumers must dose more detergent. Therefore, manufacturers have begun marketing laundry capsules of larger sizes than were previously sold.

We conducted this research to study the impact of size of laundry detergent capsules on the desire of toddlers to interact with them. Children develop gripping ability rapidly through the first decade of life, with particularly accelerated development occurring in the first 5 years of life, a developmental period that overlaps closely with a period of heightened risk for child poisoning [6–8,30–32]. There is some evidence from both infants [33] and older children [34] that children may prefer to grasp objects that fit in their hands easily, although even the larger-size laundry capsules currently on the market are easily gripped by toddler-sized hands. In the child poisoning field, one study offers preliminary data to suggest the size of bottles containing hazardous and safe household liquids may not influence toddler’s preferences for those bottles [21]. Our core research question was therefore rather straightforward: will toddlers interact more with large laundry capsule sizes or small ones?

We evaluated this question in three empirical studies, each of which implemented methodology adapted from previous research evaluating children’s preference for laundry capsules of different colours [25]. In Study 1, we considered toddler’s behaviour in a forced-choice test where they were presented with four capsules in a randomized order, two small and two large, to determine which capsule they grasped first. In Study 2, we considered toddler’s behaviour in a more ecologically valid research design with capsules accessible in the original container that was left open. Children were presented with a simulated home laundry cabinet containing multiple products, including laundry capsules, and the first three items they chose to grasp and handle were recorded. Study 3 used a similar ecologically valid design but incorporated the presence of a laundry capsule left outside of the original closed packaging, on the cabinet shelf. The container of capsules presented in Study 3 was fastened with a child resistant lid closure. Thus, the combination of Studies 2 and 3 evaluated toddler’s behaviour in the two most common risk situations, when capsules are accessible to young children in open containers and when a capsule left outside the original container. All research was conducted with toddlers aged 9–36 months, the age group at greatest risk of unintentional exposure [6–8].

## Materials and methods

### Overview of methods

The research was comprised of three studies, each investigating toddler’s preferences toward and interaction with small versus larger laundry capsules. Studies 1 and 2 were carried out in May and June 2019 and Study 3 in October through December 2019. The same participants taking part in Study 1 also took part in Study 2. A new group of toddlers was recruited for Study 3. All parents provided informed consent and toddlers provided developmentally appropriate assent. All study protocols were approved by the Ethics Committee in Human Research of the University of Valencia, Spain (Reference Number 1033674). The University of Valencia ethical committee was chosen for approval because it specialises in social research and represents the regional area where the study was conducted.

Inclusion criteria for all three studies were toddlers ages 9–36 months with a parent or legal guardian who could communicate orally in Spanish. Exclusion criteria were minimal and included any physical or mental disorder that precluded valid participation in the research. All toddlers were required to be able to stand, sit by themselves, and understand oral orders in Spanish. No toddlers were excluded from any study for these reasons.

Children were recruited through Asociación de Investigación de la Industria del Juguete **(**AIJU), a research centre that specializes in investigations about child behaviour and preferences. Toddlers were recruited for the studies using AIJU’s database of collaborating kindergartens from 5 locations in the province of Valencia, Spain. The 5 locations (Valencia, Alfafar, Xativa, Alboraia, Meliana) were selected from different districts and neighbourhoods to promote social diversity and represent mixed income. All research was conducted in a child-friendly environment in the kindergartens by the same AIJU researcher. The environment was a dedicated room with the same set up including identical replications of toddler-sized furniture, and a replicate of an open laundry room storage shelf.

All laundry capsules used in the studies were fabricated to be safe for children while appearing identical to commercially available laundry capsules. They were filled with honey, which was dyed with food-grade colouring to offer not just a similar physical appearance but also a similar tactile presentation to the actual products. The smaller capsule contained 23g of honey and had a footprint of 41 x 43mm. The larger capsule contained twice the amount of honey, representing a double dosage recommended for a large or heavily stained load of laundry of 8 pounds (3.63 kg) or more. The 46g of honey in the larger product was placed within a capsule footprint of 50 x 53mm. The capsules therefore looked and felt like marketplace products, with similar shapes, colors, and designs.

For Studies 2 and 3, original packaging and labelling of all products were used but the contents were replaced for safety with water to the correct weight.

#### Study 1 and 2 participants

Studies 1 and 2 were conducted with the same sample of toddlers. A total of 156 toddlers (mean age = 24 months, SD = 7.3; 78 boys, 78 girls, range = 9–36 months) participated. Toddlers were recruited at a 4:3 ratio across the sub-age groups of 9–24 months and 25–36 months, reflecting the age distribution of children involved in unintentional exposure scenarios reported among children [3,4,6–8]. Sample size was driven by a non-inferiority hypothesis that the larger size capsule would be non-inferior to the smaller size in terms of probability of child selection. Based on findings in related previous research [25], the proportion test method assumed the probability of choosing the smaller size capsule would be 6%, the probability of choosing the larger size capsule would be 7%, and the margin of non-inferiority would be 8%. With those assumptions, a minimum sample size of 154 children was required to achieve 80% power. Expecting experimenter error or other research protocol violations may lead to two withdrawals, we recruited 156 children for each experiment.

#### Study 1 procedure

**Fig 1:**
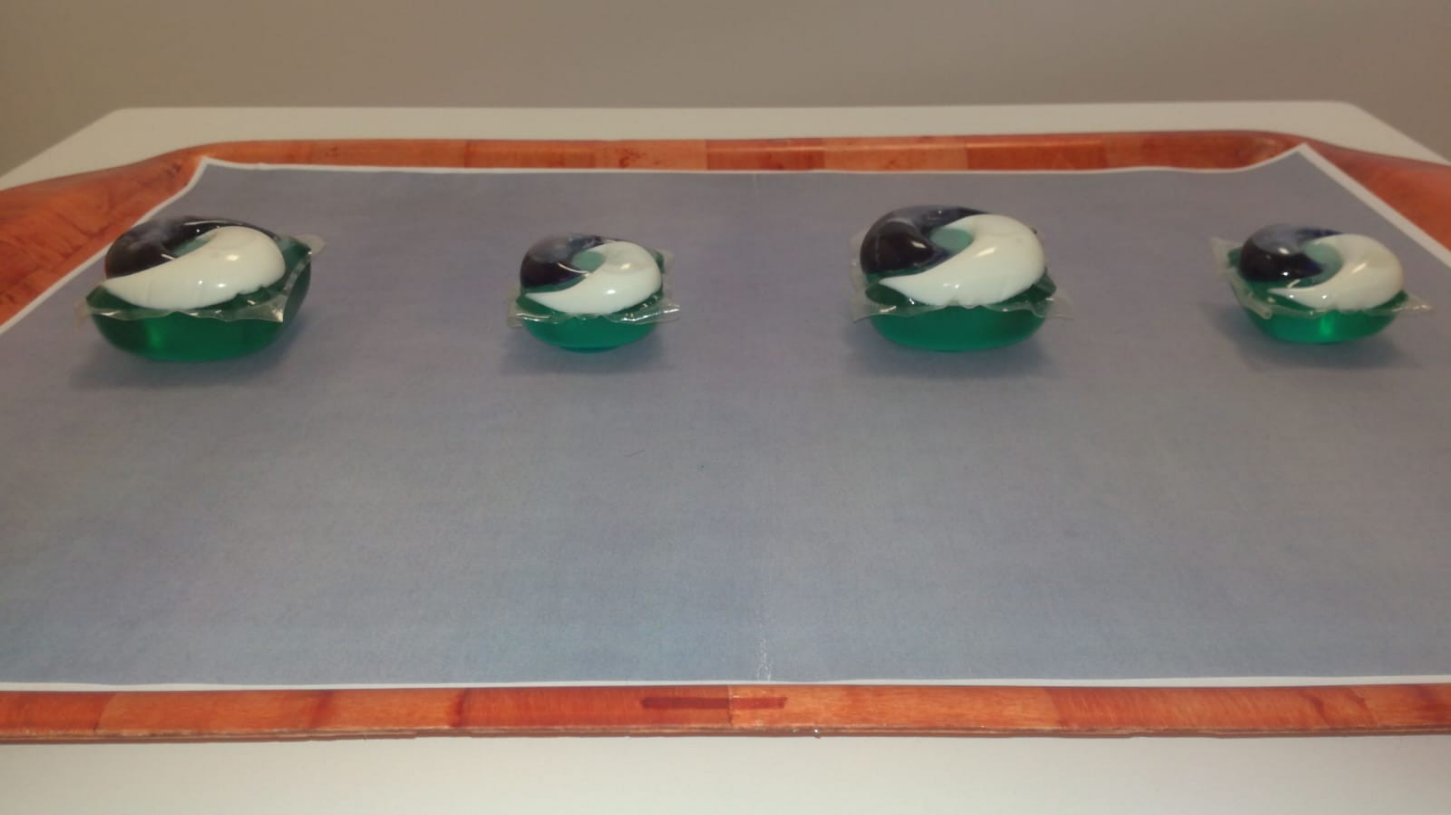
Photograph of stimuli example, as presented to toddlers in Study 1.

Study 1 was an out-of-context behavioural observation study using a forced-choice methodology. Toddlers were seated at a table and presented with a total of four replicas of two sizes of laundry capsules alongside one another in a straight line on a neutral grey background (Fig 1). Toddlers were purposely seated in the centre of the 4 capsules, at a distance where they could reach the capsules, but not easily. The sizes were presented to toddlers 6 times in total using a randomized order of presentation, such that small and large capsules appeared in different locations on different presentations. Toddlers were asked to “take the one capsule that you would most like”. The location and size of the first capsule that the child grasped and held (not just touched, to replicate real-world risk) was recorded. Before the trials started and between each trial, toddlers were asked to place their hands on their lap, allowing each trial to begin from a “neutral” starting position.

#### Study 2 procedure

**Fig 2:**
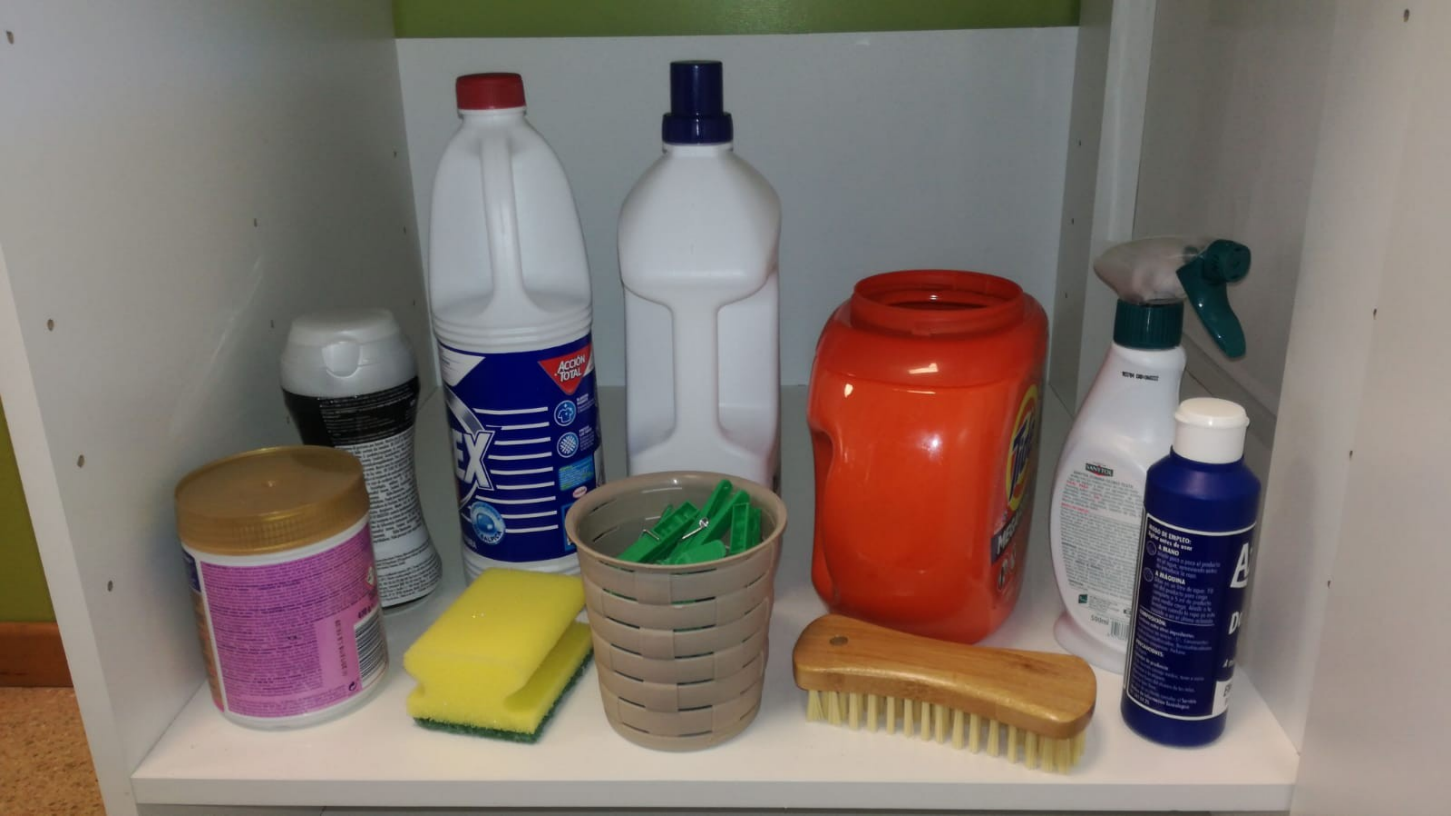
Photograph of stimuli, as presented to toddlers in Study 2.

In Study 2, the same toddlers who participated in Study 1 were presented with an ecologically valid simulated open-shelf laundry cabinet designed to mimic the storage scenario of laundry capsules alongside other household cleaning items (Fig 2). The laundry capsule container was left open, providing comparatively easy access to toddlers and representing a “real-life” risk scenario. A range of 9 common household laundry cleaning items (liquid laundry detergent, bleach, stain pre-treater in spray and tablet form, fabric enhancer in liquid and bead form, scrubbing brush, sponge, and clothes pegs) was presented in the cabinet along with the laundry capsules, which were placed inside to fill the large orange container shown in Fig 2. Laundry capsule size was randomly selected to be small or large. All products were selected to be of a size and weight that toddlers could easily grasp and handle. The arrangement and products included in the simulation were identical for all toddlers and all trials, and mimicked real-world situations in which the toddler could be at risk when taking a single capsule directly from the open container or when taking the complete open container, gaining access to many capsules.

At each visit, toddlers were allowed up to 10 minutes to play and settle in the laboratory environment. The laundry cabinet was concealed behind a screen during this time. When the researcher detected the child was comfortable, the open shelf was revealed, and the researcher asked the toddler to sit or stand in front of it and “take one thing from the shelf and give it to me”. The first three items selected were recorded.

The starting position in front of the cabinet was the same for all toddlers, and the shelf was set at a height of 30cm, providing access, visibility and reachability for all toddlers to all items. An item was considered chosen when the toddler gave it to the interviewer, or if it was fully grasped; simply touching an item did not qualify as a selection. After the first item was chosen, the researcher repeated the request for the toddler to take another item. This process was repeated three times in total. Throughout the trial, researchers were careful to not influence toddler’s choices through verbal or nonverbal gestures of any type.

The frequency with which either a single capsule or the complete container of capsules was chosen, as well as selection of the first, second and third choice, was recorded for analysis purposes.

About one week later (minimum gap of 48 hours, mean gap = 7.8 days, SD = 7.9), the same toddlers returned to the laboratory and were presented the same scenario and protocol, with the size of the laundry capsule changed. Order of size presentation was randomised across visits.

#### Study 3 participants

A total of 156 toddlers (78 boys, 78 girls; mean age = 23.4 months; SD = 6.7; range = 9–36) participated in Study 3. As in the previous studies, a 4:3 ratio across the sub age groups of 9–24 months and 25–36 months was used to replicate the age distribution of toddlers involved in incidents [3,4,6–8]. Sample size was determined using the same proportion test method as for Study 1.

#### Study 3 methods

**Fig 3:**
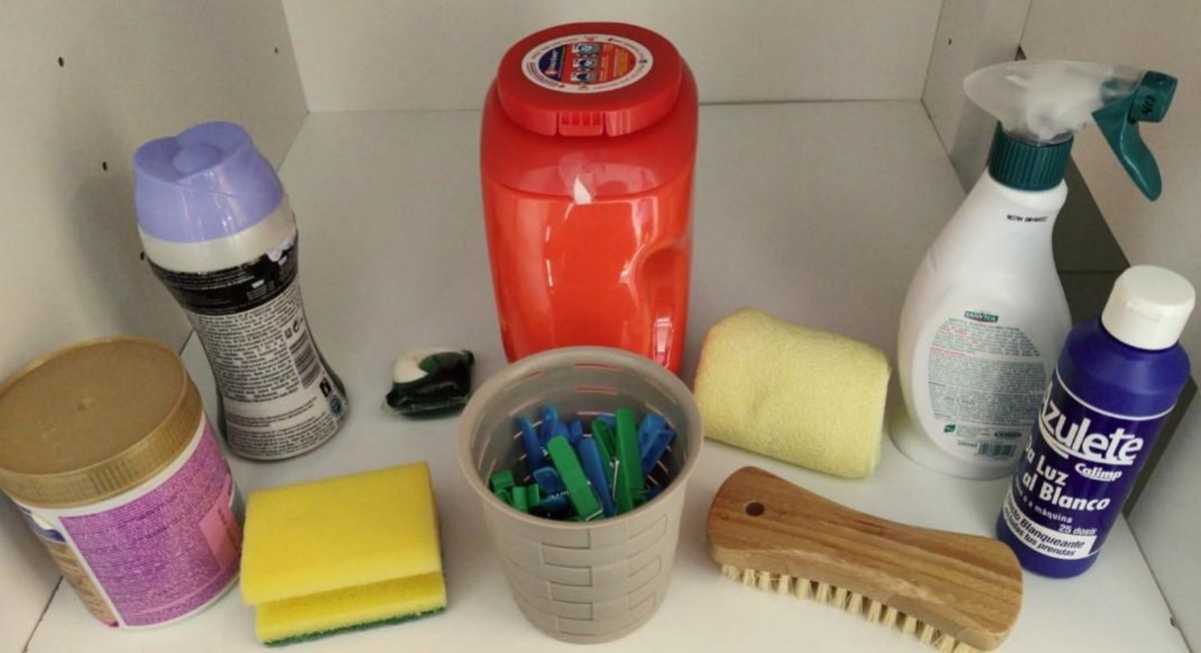
Photograph of stimuli, as presented to toddlers in Study 3.

Study 3 extended Study 2 by leaving a capsule outside the closed pack of capsules, thus offering a different situation that may present risk of unintentional access to young children. Toddlers were presented with the same ecologically valid simulated open-shelf laundry cabinet from Study 2, again designed to mimic the storage scenario of laundry capsules. Laundry capsule size was randomly selected. In Study 3, the container of laundry capsules was closed using the child-resistant fastener, but one capsule was left outside and beside the pack (Fig 3). This mimicked a situation where the child could be at risk from the exposed capsule, but minimal risk was present from the closed pack. Again, toddlers were asked three successive times by the researcher to “take one thing from the shelf and give it to me”. The first three items selected were recorded.

As in Study 2, toddlers were allowed up to 10 minutes initially to settle, and then the open shelf was revealed from behind the screen. The researcher asked the toddler to “take one thing from the shelf and give it to me”. The position in front of the cupboard was the same for all toddlers, and all items were reachable for all children. After the toddler’s selection was recorded, they were asked to give the item to their caregiver, and the item was put aside. The choice process was repeated three times in total, and the frequency with which either a single capsule or the complete container containing the capsules was chosen was calculated for first, second and third choice. The sum of the three choices was additionally calculated.

As in Study 2, the same toddlers returned to the laboratory about one week later (minimum gap of 48 hours; mean = 4.6 days, SD = 2.5) and completed the same scenario and protocol, with the size of the laundry capsule changed.

#### Statistical analyses

Preliminary testing for all three studies comparing boys vs girls and young vs older children yielded no pattern of consistent differences at statistically significant or marginal levels, so all analyses were conducted across toddler sex and age. Analyses for Study 1 were conducted by comparing selection of the two differently sized capsules using a conjoint model with likelihood ratio test in JMP 15.0 (100 SAS Campus Drive, Cary, NC 27513–2414, USA). Given the sample size, a confidence interval at 95% significance level—within (44%, 56%)—indicates the probability of choosing the small capsule is equivalent to the probability of choosing the large one. The interval of (44%, 56%) was predefined based on previous research [25].

For Study 2, twelve mixed models using the Glimmix procedure in SAS 9.4 (100 SAS Campus Drive, Cary, NC 27513–2414, USA) were computed to evaluate toddlers’ preferences, with toddler’s choice serving as response variable; product, order and product*order as fixed effects; and participant as a random effect. Similarly, for Study 3, four mixed models were computed using the Glimmix procedure in SAS 9.4 to evaluate toddlers’ preferences, again with toddler’s choice as response variable; product, order and product*order as fixed effects; and participant as a random effect. All analyses were conducted using an alpha of 0.05 as a significance level.

## Results

### Study 1 results

To evaluate toddler’s selections of the small versus large capsule in the Study 1 laboratory forced-choice test, a conditional logit model with Likelihood Ratio (LR) chi-square test was computed. The model includes toddler’s choice as the response variable, capsule size and capsule position as fixed factors, and choice set and participant as conditional grouping variables. All 936 choices were included in the model. As shown in Table 1, the small capsule was chosen 49.8% of the time while the large capsule was chosen 50.2% of the time. The LR chi-square test indicated the two capsule sizes were chosen with similar frequency (χ^2^(1) = 0.018, *p* = 0.89). The confidence interval for the selection of both capsule sizes included 50% and was smaller than (44%, 56%), indicating the probability of choosing the small size capsule and the probability of choosing the large size capsule are equivalent.

**Table 1:** Study 1: Probability of toddler choosing small versus large capsule (N = 156 toddler; n = 936 selections).

Capsule Size	Frequency of Selection	Standard Error	*p*-value	95% Confidence Interval
**Small**	49.8%	1.7%	0.89	(46.5%, 53.1%)
**Large**	50.2%			(46.9%, 53.5%)

Given this result, a conditional logit model was computed to determine whether laundry capsule position influenced toddlers’ selections rather than size. The LR chi-square value indicated that there was a statistically significant preference for toddlers to select capsules in Position 2 (centre right from child’s perspective; 42.2% of selections) more often than capsules in the other three positions. Toddlers also chose capsules in Position 4 (far left; 11.9% of selections) less often than capsules in the other three positions. These differences were statistically significant (χ^2^(3) = 175.56, *p* < .0001; See Table 2).

**Table 2:** Study 1: Probability of toddler picking capsule in each position on table (N = 156 children; n = 936 selections).

Capsule Position	Frequency of Selection	Standard Error	95% Confidence Interval
**1 –Far Right**	22.3%	1.8%	(19.1%, 25.9%)
**2 –Centre Right**	42.2%	2.1%	(38.2%, 46.3%)
**3 –Centre Left**	23.6%	1.8%	(20.3%, 27.3%)
**4 –Far Left**	11.9%	1.4%	(9.5%, 14.8%)

***Note*. The p-value for overall comparison is <0.0001. Capsule positions are expressed from the child’s perspective.**

### Study 2 results

To evaluate toddlers’ preferences in Study 2, we conducted 12 mixed models with the Glimmix procedure. Toddler’s choice served as the response variable; product, order and product*order as fixed effects; and participant as a random effect. As shown in Table 3, we considered first, second and third choice selections of the loose capsule, the open container of capsules, and the combination of either the loose capsule or the open container of capsules. We also considered any selection of the capsules in the first, second or third choice selection. Across the 12 mixed models, most confidence intervals for the difference between choosing the small and large capsule were within (-6, 6), indicating equivalence in probability to choose the small or the large capsule. In fact, just one statistically significant main effect for product emerged at a *p* < .05 level (recall that by chance, Type I error rates would lead us to expect one statistically significant result for every 20 analyses conducted); that significant result indicated a preference for toddlers to select the small loose capsule (18% of selections) more often than the large loose capsule (13% of selections) for any of their three choices (Wald χ^2^(1) = 5.5, *p* = 0.019; See Table 3). There also was just one statistically significant main effect for order across the 12 models, and no product*order effects, so we concluded order of presentation did not influence toddler’s decisions.

**Table 3:** Study 2: Percentage of toddlers choosing the container displaying the capsules, the loose capsule from inside the open container, and the sum of the container or the capsule in the first three items chosen, separated by capsule size (N = 156).

	Open Container			Capsule			Open Container or Capsule		
Capsule Size	Small % (SE)	Large % (SE)	95% CI	Small % (SE)	Large % (SE)	95% CI	Small % (SE)	Large % (SE)	95% CI
**1**^**st**^ **Choice**	3*(1*.*4)*	3(*1*.*2)*	(-3.5, 2.7)	6*(2*.*1)*	4*(1*.*5)*	(-0.8, 5.7)	9*(2*.*4)*	6*(2*.*1)*	(-1.5, 7.7)
**2**^**nd**^ **Choice**	1(*0*.*9)*	1*(0*.*9)*	(-2.5, 2.5)	7*(2*.*1)*	6*(2*.*0)*	(-3.8, 5.3)	8*(2*.*2)*	8*(2*.*1)*	(-4.2, 5.5)
**3**^**rd**^ **Choice**	4*(1*.*7)*	5*(1*.*8)*	(-4.8, 3.7)	4*(1*.*7)*	3*(1*.*3)*	(-1.4, 5.2)	9*(2*.*3)*	8*(2*.*1)*	(-4.0, 6.6)
**Sum of Choices**	8*(2*.*2)*	9*(2*.*4)*	(-5.0, 4.4)	18*(3*.*1)*	13*(2*.*7)*	(0.9, 10.3)*	27*(3*.*6)*	22*(3*.*4)*	(-1.6, 10.8)

* *p* < 0.05.

### Study 3 results

To evaluate toddlers’ preferences in Study 3, we conducted four mixed models with the Glimmix procedure. Toddler’s choice served as the response variable; product, order and product*order as fixed effects; and participant as a random effect. As shown in Table 4, we considered first, second and third choice selections of the loose capsule. We also considered any selection of the capsule in the first, second or third choice selection. Across the four mixed models, there were no statistically significant main effects at a *p* < .05 level for product choice. There also were no statistically significant effects for order or product*order in any of the models. We thus concluded neither capsule size nor order of presentation influenced toddler’s decisions.

**Table 4:** Study 3: Percentage of toddlers choosing the loose capsule, separated by capsule size (N = 156).

	Capsule		
Capsule Size	Small % (SE)	Large % (SE)	95% CI
**1**^**st**^ **Choice**	3.8*(1*.*5)*	4.4*(1*.*7)*	(-3.4, 2.2)
**2**^**nd**^ **Choice**	5.7*(1*.*9)*	5.5*(1*.*9)*	(-4.8, 5.4)
**3**^**rd**^ **Choice**	8.3*(2*.*2)*	8.6*(2*.*3)*	(-5.9, 5.2)
**Sum of Choices**	18*(3*.*1)*	19.2*(3*.*2)*	(-7.4, 4.9)

## Discussion

This research examined the impact of the size of laundry detergent capsules on toddler’s desire to interact with them. The general pattern of results suggests toddlers do not demonstrate a strong preference to interact with smaller or with larger capsules; across all studies and trials, we found a consistent pattern of no differences in their preferences or selections.

Previous research on child poisoning risk suggests young children interact with different household products at different rates, depending on factors such as the shape, labelling, and transparency of those products and the containers holding them [17–22]. Research focused on laundry detergent capsules in an ecologically valid setting similar to that used in the present study offers indication that toddlers do not show preference in grasping choice between an assortment of coloured and multi-coloured capsules [25].

The largely null results in the present study suggest the size of laundry capsules is unlikely to play a major role in child poisoning risk. Grasping of objects is a kinematic skill that develops through at least the first decade of life [30], and available evidence suggests children may prefer to grip objects that are well within their grasping ability. Research with 5- to 12-month infants, for example, found that babies preferred to reach for and handle smaller objects, which may fit in their hands more easily [33]. Research with older children supports this possibility also, as 7- and 8-year-olds perform better catching smaller rather than larger rubber balls [34]. In both those studies, however, objects ranged substantially in size. In our current study, both sizes of laundry capsules were well within the grasping ability of all children studied, creating a situation where children appeared to show no preference to grasp one size over the other. We also detected no consistent gender or age differences among our sample.

More broadly, the literature on child poisoning incidents indicates a wide range of factors contribute to individual paediatric poisoning incidents. These factors include Bronfenbrenner microsystem influences such as parent supervision and household product storage habits, macrosystem influences such as cultural factors and industry decisions about packaging and, the present focus, child factors to touch and mouth products that are unknown by the child to be dangerous [35–38]. From an ecological theory perspective, the present results offer guidance for prevention through multiple pathways, and at multiple levels of the ecological framework. From a parenting (microsystem) perspective, the results reinforce the need to store laundry capsules and other dangerous products safely and out of reach from children. From a policy (macrosystem) perspective, they reinforce the need for regulations in all jurisdictions to market laundry capsules in packaging that deters children from gaining access.

From an industry (macrosystem) perspective, laundry detergent manufacturers follow government regulations to market laundry capsules in packaging that include child-resistant or child-impeding fasteners designed to restrict or slow children’s access to the dangerous products inside. There is good evidence on the efficacy of such fasteners [39–42]. Second, manufacturers recommend to consumers that they store capsules in the original packaging, and out of reach of toddlers. Terms like “closed, up and away” [43] are reinforced frequently to consumers, both by the detergent industry [44] and by partners such as the US Centers for Disease Control [45] and non-profit entities like Safe Kids Worldwide [46].

Despite existing prevention efforts, available evidence from case studies suggests that unintentional poisoning incidents occur when children access capsules either in packs that are left open or when capsules are left outside the original packaging in places accessible to toddlers [23]. This may be because parents and other adults misunderstand or underestimate the potential risks present from laundry detergent capsules [13–16]. The present research suggests capsule size may not impact children’s interaction with capsules that are left in a location they can obtain it; previous research suggests capsule colour also plays no significant role in children’s attention to and interaction with capsules [25]. Continued research is needed to investigate whether different types or styles of capsules may improve child safety, or whether prevention efforts are best directed toward alternative strategies.

One secondary result of interest emerged from our research. Results from Study 1 suggested there may be some impact of toddler interaction with dangerous household items like laundry capsules based on the placement of those items. Results from Study 2 and Study 3 support this finding, as many children in those studies chose not to handle the laundry capsules, which were fully visible to all children but located in the back of the cabinet. In fact, post-hoc analyses revealed that children showed a greater tendency to handle items close to the front of the cabinet (e.g., the sponge was handled between 47–61% of the time depending on condition and study). This finding is not entirely surprising–one might expect toddlers to choose to handle the objects closest to them, and closest to their dominant (usually right) hand–but we recommend further research designed specifically to test these effects. If the results are replicated, they may have implications to suggest parents should store hazardous products such as laundry capsules in less prominent areas of their storage cabinets. They also suggest product location may be a more prominent risk for child poisoning incidents than product size, colour or appearance.

Like all research, our studies had limitations. The structured forced choice situation in Study 1 offered the advantage of a forced choice of one size capsule or the other but may have created a situation where children behaved different from the way they would behave in a real-world environment. Studies 2 and 3 overcame that limitation to some extent by introducing a more ecologically valid scenario, but they were still conducted outside the child’s own home and with a simulated laundry cabinet situation. We also limited our sample to children in the highest-risk age group based on epidemiological data, but future research might consider risk among younger and older children. Finally, our sample was collected from multiple geographic regions, all within one province of Spain. One might expect reasonable generalizability of the child development aspects of what we studied, but geographic generalizability is unknown.

## Conclusion

The findings from Studies 1, 2 and 3 consistently failed to demonstrate statistical differences in levels of toddler interaction measured by grasping preferences between the small and large size capsules. This was the case for Study 1, which was designed to create exaggerated conditions where the different capsules were chosen side by side in a forced-choice scenario, as well as for Studies 2 and 3, which were designed to more realistically mimic potential situations in the home where a capsule had been left accessible to a toddler. We conclude that the difference in size studied in this research, representing small and large size capsules, is not expected to impact toddler interaction rates with dangerous laundry detergent capsules in the home.

## Supporting information

S1 Data Data study 1. (XLSX)

S2 Data Data study 2. (XLSX)

S3 Data Data study 3. (XLSX)

## References

[1] Kekatos M. Laundry pods poisoning kids–new designs look tastier. Daily Mail Online. 2 February 2017 [cited Jan 27, 2020]. Available from http://www.dailymail.co.uk/health/article-4185644/Laundry-pods-poisoning-kids-new-designs-look-tastier.html.

[2] Mohney G. Detergent pods remain a danger for young children, study finds. ABC News. 25 April 2016 [cited Jan 27, 2020]. Available from: http://abcnews.go.com/Health/detergent-pods-remain-danger-young-children-study-finds/story?id=38654774.

[3] Swain TA, McGwin G, Griffin R. Laundry pod and non-pod detergent related emergency department visits occurring in children in the USA. Inj Prev. 2016;22(6):396–399. 10.1136/injuryprev-2016-041997 27339062

[4] Valdez AL, Casavant MJ, Spiller HA, Chounthirath T, Xiang HY, Smith GA. Pediatric exposure to laundry detergent pods. Pediatrics. 2014;134(6):1127–1135. 10.1542/peds.2014-0057 25384489

[5] Rigaux-Barry F, Patat AM, Cordier L, Manel J, Sinno-Tellier S. Risks related to pods exposure compared to traditional laundry detergent products: study of cases recorded by French PCC from 2005 to 2012. Toxicologie Analytique Et Clinique. 2017;29(3):257–266.

[6] Settimi L, Giordano F, Lauria L, Celentano A, Sesana F, Devanzo F. Surveillance of paediatric exposures to liquid laundry detergent pods in Italy. Inj Prev. 2019;24:5–11. 10.1136/injuryprev-2016-042263 PMC5800340 28188147

[7] World Health Organization. World Report on Child Injury Prevention. 2008. Available from: https://www.who.int/violence_injury_prevention/child/injury/world_report/en/. 26269872

[8] International Association for Soaps, Detergents and Maintenance Products (A.I.S.E.) Detergent capsules “Accidentology” project final report. 2015 [cited Jan 27, 2020]. Available from https://www.aise.eu/documents/document/20151103103823-microsoft_word_-_detergent_capsules_accidentology_-_final_report_2nov2015.pdf.

[9] Gibbs L, Waters E, Sherrard J, Ozanne-Smith J, Robinson J, Young S, et al Understanding parental motivators and barriers to uptake of child poison safety strategies: a qualitative study. Inj Prev. 2005;11(6):373–377. 10.1136/ip.2004.007211 16326774 PMC1730291

[10] Schwebel DC, Evans WD, Hoeffler SE, Marlenga BL, Nguyen SP, Jovanov E, et al Unintentional child poisoning risk: a review of causal factors and prevention studies. Child Health Care. 2017;46(2):109–130.

[11] Bronfenbrenner U. Toward an experimental ecology of human development. Amer Psychol. 1977;32(7):513–531.

[12] Bearth A, Miesler L, Siegrist M. Consumers’ risk perception of household cleaning and washing products. Risk Anal. 2017;37(4):647–660. 10.1111/risa.12635 27163359

[13] Bearth A, Buchmüller K, Bürgy H, Siegrist M. Barriers to the sae use of chemical household products: a comparison across European countries. Environ Res. 2019;180: 108859 10.1016/j.envres.2019.108859 31706596

[14] Beirens TMJ, van Beeck EF, Dekker R, Brug J, Raat H. Unsafe storage of poisons in homes with toddlers. Acc Anal Prev. 2006;38(4):772–776. 10.1016/j.aap.2006.02.007 16545327

[15] Buchmüller K, Bearth A, Siegrist M. Consumers’ perceptions of chemical household products and the associated risks. Food Chem Toxicol. 2020;143:111511 10.1016/j.fct.2020.111511 32610062

[16] Patel B, Devalia B, Kendrick D, Groom L. Validating parents’ self-reports of children’s exposure to potentially toxic substances within the home. Inj Prev. 2008;14(3):170–175. 10.1136/ip.2007.017780 18523109

[17] Basso F, Bouillé J, Le Goff K, Robert-Demontrond P, Oullier O. Assessing the role of shape and label in the misleading packaging of food imitating products: from empirical evidence to policy recommendation. Front Psych. 2016;7:450. 10.3389/fpsyg.2016.00450 PMC4814518 27065919

[18] Jones SS, Smith LB. How children know the relevant properties for generalizing object names. Dev Sci. 2002;94:219–232.

[19] Macario JF. Young children’s use of color in classification: foods and canonically colored objects. Cog Dev. 1991;6(1): 17–46.

[20] Pereira AF, Smith LB. Developmental changes in visual object recognition between 18 and 24 months of age. Dev Sci. 2009;12(1):67–80. 10.1111/j.1467-7687.2008.00747.x 19120414 PMC2888029

[21] Schwebel DC, Wells H, Johnston A. Children’s recognition of dangerous household products: child development and poisoning risk. J Ped Psychol. 2014;40(2):238–250. 10.1093/jpepsy/jsu088 25306403

[22] Smith LB. Learning to recognize objects. Psychol Sci. 2003;14(1):244–250. 10.1111/1467-9280.03439 12741748

[23] Day R, Bradberry SM, Thomas SHL, Vale A. Liquid laundry detergent capsules (PODS): a review of their composition and mechanisms of toxicity, and of the circumstances, routes, features, and management of exposure. J Clin Tox. 2019; 57(11):1053–1063. 10.1080/15563650.2019.1618466 31130018

[24] Williams H, Jones S, Wood K, Scott RAH, Eddleston M, Thomas SHL, et al Reported toxicity in 1486 liquid detergent capsule exposures to the UK National Poisons Information Service 2009–2012, including their ophthalmic and CNS effects. J Clin Tox. 2013;52(2):136–140. 10.3109/15563650.2013.855315 24199643

[25] Richmond A, Liang Z, Mulaj V, Ryckmans J, Stijntjes G. The importance of an ecologically valid method in the evaluation of toddler interaction with coloured liquid laundry detergent capsules. PLoS ONE. 2018;13(7):e0199976 10.1371/journal.pone.0199976 29966022 PMC6028113

[26] Yin S, Colvin J, Behrman A. Single-use laundry detergent pack exposures in children under 6 years: a prospective study at U.S. poison control centers. J Emerg Med. 2018;55:354–365. 10.1016/j.jemermed.2018.05.016 29941372

[27] A.I.S.E. Pan-European consumer habits survey 2018 [Internet]. A.I.S.E. Available from https://www.aise.eu/documents/document/20180528165059-aise_consumershabitssurvey2017_summary_final.pdf.

[28] Department of Energy. Energy and Water Conservation Standards, 10 C.F.R. [2019].

[29] Association of Home Appliance Manufacturers (AHAM). Clothes Washers Energy Efficiency and Consumption Trends, 1990–2015. Washington, DC.

[30] Kuhtz-Buschbeck J, Stolze H, Johnk K, Boczek-Funcke A, Illert M. Development of prehension movements in children: a kinematic study. Exp Brain Res. 1998;122:424–432. 10.1007/s002210050530 9827861

[31] Vaivre-Douret L, Burnod Y. Development of a global motor rating scale for young children (0–4 years) including eye-hand grip coordination. Child Care Hlth Dev. 2008;27(6):515–534. 10.1046/j.1365-2214.2001.00221.x 11737019

[32] Zoia S, Pezzetta E, Blason L, Scabar A, Carrozzi M, Bulgheroni M, et al A comparison of the reach-to-grasp movement between children and adults: a kinematic study. Dev Neuropsychol. 2006;30(2):719–738. 10.1207/s15326942dn3002_4 16995833

[33] Libertus K, Gibson J, Hidayatallah N, Hirtle J, Adcock RA, Needham A. Size matters: how age and reaching experiences shape infants’ preferences for different sized objects. Infant Behav Dev. 2013;36(2):189–198. 10.1016/j.infbeh.2013.01.006 23454420 PMC3757549

[34] Isaacs L. Effects of ball size, ball color, and preferred color on catching by young children. Percept Mot Skills. 1980;51:583–586. 10.2466/pms.1980.51.2.583 7443379

[35] Schwebel D, Wells H, Johnston A. Children’s recognition of dangerous household products: child development and poisoning risk. J Ped Psychol. 2015;40(2):238–250. 10.1093/jpepsy/jsu088 25306403

[36] Schwebel D, Douglas Evans W, Hoeffler S, Marlenga B, Nguyen S, Jovanov E, et al Unintentional child poisoning risk: a review of causal factors and prevention studies. Child Health Care. 2017;46(2):109–130.

[37] Soori H. Developmental risk factors for unintentional childhood poisoning. Saudi Med J. 2001;22(3):227–230. 11307108

[38] Meyer S, Eddleston M, Bailey B, Desel H, Gottschling S, Gortner L. Unintentional household poisoning in children. Klin Pädiatr. 2007;219(5):254–270. 10.1055/s-2007-972567 17763291

[39] Krug A, Ellis JB, Hay IT, Mokgabudi NF, Robertson J. The impact of child-resistant containers on the incidence of paraffin (kerosene) ingestion in children. *S Afr Med J*. 1994;84(11):730–734. 7495007

[40] US Consumer Product Safety Commission. Poison prevention packaging: A guide for healthcare professionals. Washington, DC, 2005. https://www.cpsc.gov/s3fs-public/pdfs/blk_media_384.pdf.

[41] Rodgers GB. The effectiveness of child-resistant packages for aspirin. Arch Pediatr Adolesc Med. 2002;156:929–933. 10.1001/archpedi.156.9.929 12197802

[42] Rodgers GB. The safety effects of child-resistant packaging for oral prescription drugs. J Amer Med Assoc. 1996;275(21):1661–1665. 8637140

[43] PROTECT. Put your medicines up and away and out of sight [Internet]. PROTECT; 2017. Available from https://www.upandaway.org/.

[44] Tide. At Tide safety comes first and it never stops [Internet]. Tide; 2020. Available from: https://tide.com/en-us/safety.

[45] CDC. Put your medicines *up and away* and out of sight [Internet]. Up and Away Campaign; 2017 Oct 30. Available from: https://www.cdc.gov/medicationsafety/protect/campaign.html.

[46] Safe Kids Worldwide. Where do you store your medicine? [Internet]. Safe Kids Worldwide [2017 Mar 14]. Available from https://www.safekids.org/blog/where-do-you-store-your-medicine.

